# Separation and Identification of Four New Compounds with Antibacterial Activity from *Portulaca oleracea* L.

**DOI:** 10.3390/molecules200916375

**Published:** 2015-09-10

**Authors:** Xia Lei, Jianmin Li, Bin Liu, Ning Zhang, Haiyang Liu

**Affiliations:** Basic research department of Jiamusi College, Heilongjiang University of Chinese Medicine, No. 39 Guang Hua Street, Qian Jin District, Jia Mu Si 154100, China; E-Mails: leixia2006@163.com (X.L.); jmsxyrsk@163.com (J.L.); liubin169@163.com (B.L.); zhangning345678@163.com (N.Z.)

**Keywords:** *Portulaca oleracea* L., antibacterial activity, ceramides, cerebrosides, enteropathogenic bacteria

## Abstract

The *Portulaca oleracea* L. (*P*. *oleracea*) has been used to treat bacillary dysentery for thousands of years in China. Pharmacology studies on *P*. *oleracea* have also showed its significant antibacterial effects on the enteropathogenic bacteria, which might reveal the treatment of *P*. *oleracea* in cases of bacillary dysentery to some extent. To date, however, the therapeutic basis of *P*. *oleracea* treating on bacillary dysentery remains unknown. We determined the antibacterial effective fraction of *P*. *oleracea* in a previous study. The current study, which is based on our previous study, was first designed to isolate, identify and screen antibacterial active constituents from *P*. *oleracea*. As a result, four new compounds (**1**–**4**), portulacerebroside B (**1**), portulacerebroside C (**2**), portulacerebroside D (**3**) and portulaceramide A (**4**) along with five known compounds (**5**–**9**) were isolated, and structures were established by their physico-chemical constants and spectroscopic analysis. The antibacterial activities against common enteropathogenic bacteria were evaluated for all compounds and the new compounds **1**–**4** showed significant antibacterial effect on enteropathogenic bacteria *in vitro*, which might contribute to revealing the treatment of *P*. *oleracea* in cases of bacillary dysentery.

## 1. Introduction

The genus of *Portulaca* is an annual herb which taxonomically belongs to the family of *Portulacaceae*. Although it originates from India, *Portulaca* has been widely distributed in other temperate and tropical areas of the world [1,2]. In China, there are six species of *Portulaca*, among which *Portulaca oleracea* L. (*P*. *oleracea*) has been used as a traditional Chinese medicine (TCM) for thousands of years. *P*. *oleracea*, cold in nature and acid in flavor, possesses the efficacies of *clearing away the heat evil* and *detoxifying and cooling blood to stop diarrhea*. In clinical situations, *P*. *oleracea* has been used to treat acute appendicitis, scrofula ulcer, pediatric pertussis, burns and scalds, psoriasis [3], hemorrhinia, uterine bleeding, urinary tract infections, lung abscess, mumps, and especially more effective in bacillary dysentery which manifested feeling cold or fever, bellyache, diarrhea, tenesmus, and mucus pus blood stool [2]. Chemical studies on *P*. *oleracea* showed its main constituents of fatty acids, terpenes, alkaloids, coumarins, flavonoids, and volatile oil [2]. In addition, the only cerebroside of portulacerebroside A was reported in 2008 [4]. Pharmacology studies on *P*. *oleracea* showed its activities of antibacterial [5,6], hepatoprotective [7,8], anti-inflammatory, analgesia [2,9], muscle relaxant [10], neuroprotective [11], anti-oxidant [1], and anti-aging [12], but studies on the corresponding therapeutic basis were far from sufficient. Obviously, the treatment of *P*. *oleracea* on bacillary dysentery results from its antibacterial effect on the enteropathogenic bacteria; however, the corresponding active components in *P*. *oleracea* were unknown. Consequently, we screened and obtained the antibacterial fraction (EtOAc extract) from *P*. *oleracea*. The effective fraction could inhibit and kill the common enteropathogenic bacteria *in vitro* effectively, based on which a bioassay-guided isolation and phytochemical study of *P*. *oleracea* was performed and four new along with five known compounds were obtained from the effective fraction. The structures of known compounds **5**–**9** were determined by detailed 1D- and 2D-NMR analyses, ESI-MS and comparison of their spectral data with literature values was undertaken. In this paper, the isolation and structural elucidation of the new compounds **1**–**4** was described. We also investigated the antibacterial effects of compounds **1**–**9** against common enteropathogenic bacteria *in vitro*.

## 2. Results and Discussion

Compound **1** was obtained as white amorphous powder, ${\left[\alpha \right]}_{D}^{22}$ = +13.3° (*c* = 0.52, C_5_H_5_N). The molecular formula C_39_H_75_NO_9_ was established for **1** by HRESIMS *m*/*z* 702.5516 [M + H]^+^ (calc. for 702.5520), indicating three degrees of unsaturation. Methanolysis experiment of **1** liberated D-glucose suggested that **1** was a D-glucoside. The IR absorption bands at 3420 cm^−1^, 1645 and 1542 cm^−1^, and 722 cm^−1^ originated from hydroxyl, amide, and methylene groups, respectively.

**Figure 1:**
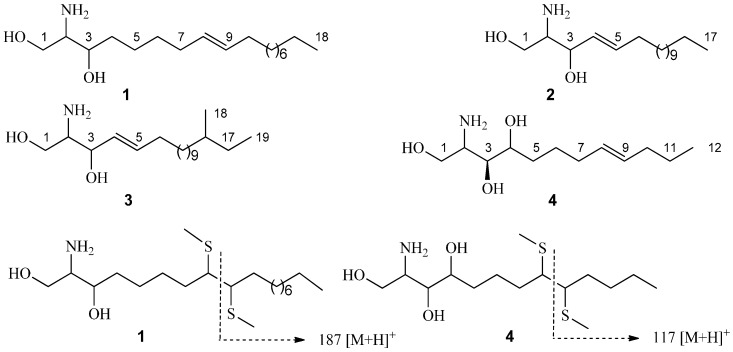
GC-MS analysis long-chain base (LCB) **1**–**4** and dimethyl disulfide (DMDS) derivatives of LCBs from **1** and **4**.

**Figure 2:**
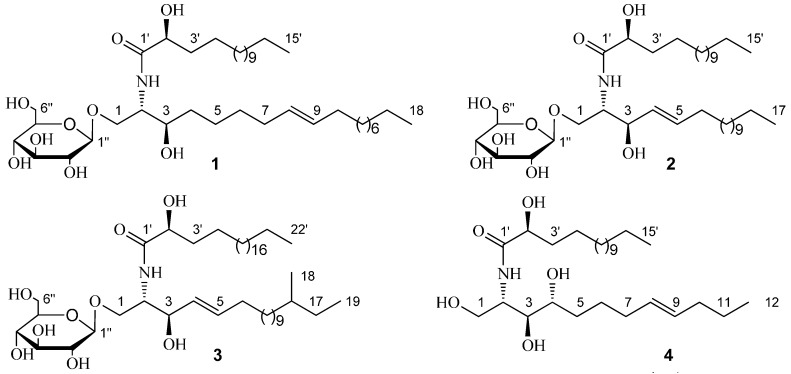
Structures of compounds **1**–**4**.

The ^1^H- and ^13^C-NMR data (anomeric proton δ_H_ 4.90, 1H, d, *J* = 7.6 Hz; δ_C_ 105.7, 75.2, 78.5, 71.6, 78.6, and 62.7) of **1** suggested the presence of a β-d-glucopyranosyl moiety. The ^1^H- and ^13^C-NMR spectra (Table 1 and Table 2) showed characteristics of a cerebroside with a 2-hydroxy fatty acid fraction as the aglycone of **1**. Methanolysis of **1** obtained a fatty acid methyl ester (FAME) and a long-chain base (LCB). The FAM was determined as 2-hydroxypentadecanoic acid methyl ester by Gas Chromatography-Mass Spectrometer (GC-MS) analysis. The 1D-TOCSY spectrum of **1** showed a correlation between δ_H_ 4.22 (1H, m, H-3) and 5.46 (2H, m, H-8, 9), which suggested the olefinic bond was located in the LCB. To determine the location of the olefinic bond in LCB, the dimethyl disulfide (DMDS) derivatives of the LCB was analyzed by ESIMS and characteristic fragment ion of *m*/*z* 187 [M + H]^+^ was obtained. Therefore, the olefinic bond was located at C-8 and C-9. The LCB was further determined as 2-aminooctadec-8-ene-1,3-diol by GC-MS analysis (Figure 1). The specific rotation ${\left[\alpha \right]}_{D}^{22}$ = −5.8° (*c* = 0.02, CHCl_3_) of the FAM confirmed that the absolute configuration of C-2′*R* [13]. The 2*S*, 3*R* stereochemistry was determined by comparing of the ^13^C-NMR data of C-2 and C-3 with those in references [14,15,16]. The *trans*-configuration (*E*) of the olefinic bond in **1** was determined by signals at δ_C_ 33.2/32.1 of the two carbons next to the olefinic bond in ^13^C-NMR spectrum [9]. The ^1^H- and ^13^C-NMR data (Table 1 and Table 2) were further assigned by the spectra of DEPT, HSQC, ^1^H-^1^H COSY, and HMBC. Consequently, **1** was established as 1-*O*-β-d-glucopyranosyl-(2*S*,3*R*,8*E*)-2-[(2′*R*)-2-hydroxylpentadecanoylamino]-8-octadecene-1,3-diol which is named as portulacerebroside B (Figure 2).

**Table 1:** ^1^H-NMR data of compounds **1**–**4** (400MHz, δ in ppm, *J* in Hz, in C_5_D_5_N-*d*_5_ at 30 °C).

1		2		3		4	
H	δ_H_ (*J*, Hz)	H	δ_H_ (*J*, Hz)	H	δ_H_ (*J*, Hz)	H	δ_H_ (*J*, Hz)
NH	8.40, d (8.4)	NH	8.36, d (8.0)	NH	8.36, d (8.0)	NH	8.55, d (8.0)
1	4.35, dd (11.6, 5.6) 4.73, m	1	4.22, m 4.72, m	1	4.22, m 4.72, m	1	4.49, dd (11.0, 4.6) 4.41, dd (11.0, 4.8)
2	4.60, m	2	4.78, m	2	4.78, m	2	5.10, m
3	4.22, m	3	4.76, m	3	4.76, m	3	4.34, dd (4.8, 6.4)
4–6	1.15–1.40, brs	4	5.86, m	4	5.86, m	4	4.27, m
7	2.10, m	5	5.98, m	5	5.98, m	5	2.22, 1.96, m
8	5.46, m	6	2.05, m	6	2.05, m	6	1.67, m
9	5.46, m	7–16	1.16–1.42, brs	7–17	1.16–1.42, brs	7	2.01, m
10	2.02, m	17	0.88, t (6.4)	18	0.88, d (7.4)	8	5.52, m
11–17	1.15–1.40, brs	2′	4.60, m	19	0.86, t (6.4)	9	5.52, m
18	0.85, t (6.4)	3′	1.86, m	2′	4.60, m	10	1.89, m
2′	4.70, m	4′	1.73, m 1.16–1.42, brs	3′	1.86, m	11	1.26–1.38, brs
3′	1.86, m	5′–14′	1.16–1.42, brs	4′	1.73, m 1.16–1.42, brs	12	0.88, t (6.8)
4′	1.73, m 1.15–1.40, brs	15′	0.88, t, 6.4	5′–21′	1.16–1.42, brs	2′	4.60, dd (7.6, 3.2)
5′–14′	1.15–1.40, brs	1′′	4.90, d, (7.6)	22′	0.88, t (6.4)	3′	2.18, 2.02, m
15′	0.85, t (6.4)	2′′	4.01, m	1′′	4.90, d, (7.6)	4′	1.96, 1.73, m
1′′	4.90, d (7.6)	3′′	4.22, m	2′′	4.01, m	5′–14′	1.26–1.38, brs
2′′	3.91, m	4′′	4.22, m	3′′	4.22, m	15′	0.88, t (6.8)
3′′	4.20, m	5′′	3.89, m	4′′	4.22, m		
4′′	4.03, m	6′′	4.34, 4.49, m	5′′	3.89, m		
5′′	4.12, m			6′′	4.34, 4.49, m		
6′′	4.18, 4.52, m						

**Table 2:** ^13^C-NMR data of compounds **1**–**4** (100 MHz, δ in ppm, in C_5_D_5_N-*d*_5_ at 30 °C).

1		2		3		4	
C	δ_C_	C	δ_C_	C	δ_C_	C	δ_C_
1	70.4, CH_2_	1	70.2, CH_2_	1	70.4, CH_2_	1	62.0, CH_2_
2	54.6, CH	2	54.6, CH	2	54.5, CH	2	52.9, CH
3	71.3, CH	3	72.4, CH	3	72.4, CH	3	76.8, CH
4–6	29.5–30.4, CH_2_	4	131.6, CH	4	131.7, CH	4	72.4, CH
7	33.2	5	132.8, CH	5	132.8, CH	5	33.8, CH_2_
8	130.2, CH	6	34.2, CH_2_	6	34.2, CH_2_	6	27.6, CH_2_
9	130.7, CH	7–14	29.6–30.4, CH_2_	7–15	29.6–30.5, CH_2_	7	33.3, CH_2_
10	32.1, CH_2_	15	32.1, CH_2_	16	35.6, CH	8	130.6, CH
11–15	29.5–30.4, CH_2_	16	22.8, CH_2_	17	30.6, CH_2_	9	130.6, CH
16	32.1, CH_2_	17	14.2, CH_3_	18	19.6, CH_3_	10	33.0, CH_2_
17	22.9, CH_2_	18		19	11.7, CH_3_	11	30.4–29.5, CH_2_
18	14.3, CH_3_	1′	175.8, C	1′	175.8, C	12	14.3, CH_3_
1′	175.5, C	2′	72.6, CH	2′	72.6, CH	1′	175.1, C
2′	72.4, CH	3′	35.7, CH_2_	3′	35.7, CH_2_	2′	72.8, CH
3′	35.7, CH_2_	4′	26.3, CH_2_	4′	26.3, CH_2_	3′	35.6, CH_2_
4′	26.3, CH_2_	5′–12′	29.6–30.4, CH_2_	5′–19′	29.6–30.4, CH_2_	4′	26.8, CH_2_
5′–12′	29.5–30.4, CH_2_	13′	32.1, CH_2_	20′	32.1, CH_2_	5′–12′	30.4–29.5, CH_2_
13′	32.1, CH_2_	14′	22.8, CH_2_	21′	22.8, CH_2_	13′	32.1, CH_2_
14′	22.9, CH_2_	15′	14.2, CH_3_	22′	14.2, CH_3_	14′	22.8, CH_2_
15′	14.3, CH_3_	1′′	105.6, CH	1′′	105.6, CH	15′	14.3, CH_3_
1′′	105.7, CH	2′′	75.1, CH	2′′	75.1, CH		
2′′	75.2, CH	3′′	78.6, CH	3′′	78.6, CH		
3′′	78.5, CH	4′′	71.5, CH	4′′	71.5, CH		
4′′	71.6, CH	5′′	78.6, CH	5′′	78.6, CH		
5′′	78.6, CH	6′′	62.6, CH_2_	6′′	62.6, CH_2_		
6′′	62.7, CH_2_						

Compound **2** was obtained as white amorphous powder, ${\left[\alpha \right]}_{D}^{22}$ = +14.8° (*c* = 0.40, C_5_H_5_N). The molecular formula was established as C_38_H_73_NO_9_ by HRESIMS *m*/*z* 688.5358 [M + H]^+^ (calc. for 688.5364), indicating three degrees of unsaturation. Methanolysis experiment of **2** liberated D-glucose suggested that **2** was a D-glucoside. The IR absorption bands at 3405 cm^−1^, 1634 and 1536 cm^−1^, and 718 cm^−1^ originated from hydroxyl, amide, and methylene groups, respectively. 

The ^1^H- and ^13^C-NMR data of compound **2** were similar with **1**, including the signals of a β-d-glucopyranosyl moiety. The position of olefinic bond in **2** was located at C-4 and C-5 by the spectra of HSQC, ^1^H-^1^H COSY, and HMBC. Methanolysis of **2** also obtained an FAM and an LCB. The FAM was determined as 2-hydroxypentadecanoic acid methyl ester by GC-MS analysis. The LCB of **2** was determined as 2-aminoheptadecenoic-4-ene-1,3-diol by GC-MS analysis (Figure 1). The specific rotation ${\left[\alpha \right]}_{D}^{22}$ = −6.8° (*c* 0.03, CHCl_3_) of the FAM confirmed that the absolute configuration of C-2′*R*, which is the same with **1**. The 2*S*, 3*R* stereochemistry was determined by comparing of the ^13^C-NMR data of C-2 and C-3 with those of in references [17,18]. The *trans*-configuration (*E*) of the olefinic bond in **2** was determined by C-6 signal at δ_C_ 34.2 in ^13^C-NMR spectrum [16]. The ^1^H- and ^13^C-NMR data (Table 1 and Table 2) were further assigned by the spectra of DEPT, HSQC, ^1^H-^1^H COSY, and HMBC. Consequently, **2** was established as 1-*O*-β-d-glucopyranosyl-(2*S*,3*R*,4*E*)-2-[(2′*R*)-2-hydroxylpentadecanoylamino]-4-heptadecene-1,3-diol which is named as portulacerebroside C (Figure 2).

Compound **3** was obtained as white amorphous powder, ${\left[\alpha \right]}_{D}^{22}$ = +8.2° (*c* = 0.15, C_5_H_5_N). The molecular formula C_47_H_91_NO_9_ was established for **3** by HRESIMS *m*/*z* 814.6765 [M + H]^+^ (calc. for 814.6772), indicating three degrees of unsaturation. Methanolysis experiment of **3** liberated D-glucose suggested that **3** was a D-glucoside. The IR absorption bands at 3412 cm^−1^, 1638 and 1530 cm^−1^, and 722 cm^−1^ originated from hydroxyl, amide, and methylene groups, respectively. 

The ^1^H- and ^13^C-NMR data of compound **3** were similar with **2** and the main difference lies in the methenyl group signal at δ_C_ 35.6 and methyl group signals at δ_C_ 11.7 and 19.6 in ^13^C-NMR spectrum of **3**, which indicated the presence of a branched methyl group in **3**. To determine the position of the branched methyl group, the 1D-TOCSY spectrum was used and correlations of δ_H_ 4.22 (1H, m, H-1) with 5.86 (1H, m, H-4), 0.88 (3H, d, *J* = 6.4 Hz), and 0.86, (3H, t, *J* = 6.4 Hz, 19-CH_3_) could be observed. Therefore, the branched methyl group was located in the LCB. Other methods to determine the structure of **3** were the same with **2**. The ^1^H- and ^13^C-NMR data (Table 1 and Table 2) were further assigned by the spectra of DEPT, HSQC, ^1^H-^1^H COSY, and HMBC. Consequently, **3** was established as 1-*O*-β-d-glucopyranosyl-(2*S*,3*R*,4*E*)-2-[(2′*R*)-2-hydroxyldocosanamideamino]-16-methyl-4-nonadecene-1,3-diol which is named as portulacerebroside D (Figure 2).

Compound **4** was obtained as white amorphous powder, ${\left[\alpha \right]}_{D}^{22}$ = +9.5° (*c* = 0.30, C_5_H_5_N). The molecular formula C_27_H_53_NO_5_ was established for **1** by HRESIMS *m*/*z* 472.3995 [M + H]^+^ (calc. for 472.4002), indicating two degrees of unsaturation. The IR absorption bands at 3401 cm^−1^, 1621 and 1533 cm^−1^, and 723 cm^−1^ originated from hydroxyl, amide, and methylene groups, respectively.

The ^1^H- and ^13^C-NMR spectra (Table 1 and Table 2) of **4** showed characteristics of a sphingosine-type ceramide with a 2-hydroxy fatty acid fraction. Methanolysis of **4** obtained an FAM and an LCB. The FAM was determined as 2-hydroxypentadecanoic acid methyl ester by GC-MS analysis. The 1D-TOCSY spectrum of **4** showed a correlation between δ_H_ 4.27 (1H, m, H-4) and 5.52 (2H, m, H-8,9), which suggested the olefinic bond was located in the LCB. To determine the location of the olefinic bond in LCB, the DMDS derivatives of LCB was analyzed by ESIMS and characteristic fragment ion of *m*/*z* 117 [M + H]^+^ was obtained. Therefore, the olefinic bond was located at C-8 and C-9. The LCB was further determined as 2-aminododecane-8-ene-1,3,4-triol by GC-MS analysis (Figure 1). The specific rotation ${\left[\alpha \right]}_{D}^{22}$ = −4.7° (*c* 0.02, CHCl_3_) of the FAM confirmed that the absolute configuration of C-2′*R* [13]. The 2*S*, 3*S*, and 4*R* stereochemistry was determined by comparing of the ^13^C-NMR data of C-2, C-3, and C-4 with those of in reference [19]. The *trans*-configuration (*E*) of the olefinic bond in **4** was determined by signals at δ_C_ 33.3/33.0 of the two carbons next to the olefinic bond in ^13^C-NMR spectrum [16]. The ^1^H- and ^13^C-NMR data (Table 1 and Table 2) were further assigned by the spectra of DEPT, HSQC, ^1^H-^1^H COSY, and HMBC and **4** was established as (2*S*,3*S*,4*R*,8*E*)-2-[(2′*R*)-2-hydroxylpentadecanoylamino]-8-dodecene-1,3,4-triol which is named as portulaceramide A (Figure 2).

**Figure 3:**
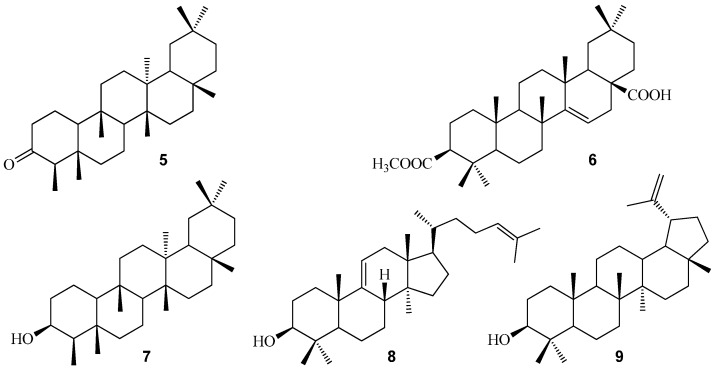
Structures of compounds **5**–**9**.

The known compounds were identified as friedelin (**6**) [20], 3-acetylaleuritolic acid (**7**) [21], 4α-methyl-3β-hydroxylfriedelan (**8**) [22], cycloartenol (**9**) [23], and lupeol (**10**) [24] by comparing their NMR spectroscopic and physical data with the literature values (Figure 3).

The antibacterial activities of compounds **1**–**9** against *Escherichia coli* (*E. coli*), *Staphylococcus aureus* (*S. aureus*), *Shigella flexneri* (*S. flexneri*), and *Salmonella typhi* (*S. typhi*) were investigated. Minimal inhibitory concentrations (MICs) and minimal bactericidal concentrations (MBCs) were determined. Compounds **1**–**4** showed significant antibacterial activity on common enteropathogenic bacteria *in vitro* (Table 3), while other compounds did not show any antibacterial effects on the enteropathogenic bacteria at the tested concentration (data not shown). The structures of compounds **1**–**4** involve a sphingoid base and an amide-linked fatty acyl chain. The amphipathic molecules exhibit diverse biological activity, including broad antibacterial activities against both Gram positive and negative bacteria in this study. 

**Table 3:** The minimal inhibitory concentrations (MICs) (mg·mL^−1^) and minimal bactericidal concentrations (MBCs) (mg·mL^−1^) of compounds **1**–**4**.

Strains	MICs (SD = 0)					MBCs (SD = 0)				
	1 *	2 *	3 *	4 *	Amoxicillin	1	2	3	4	Amoxicillin
*E. coli*	0.1875	0.1875	0.1875	0.375	2.34 × 10^−2^	0.25	0.25	0.25	0.50	3.12 × 10^−2^
*S. aureus*	0.1875	0.1875	0.1875	0.375	1.17 × 10^−2^	0.50	0.50	0.50	0.50	1.56 × 10^−2^
*S. flexneri*	0.1875	0.1875	0.1875	0.1875	5.85 × 10^−3^	0.25	0.25	0.25	0.25	3.90 × 10^−3^
*S. typhi*	0.1875	0.1875	0.1875	0.375	1.17 × 10^−2^	0.25	0.25	0.25	0.50	7.80 × 10^−3^

Key: Effects of the tested compounds on enteropathogenic bacteria. MICs and MBCs were expressed as mean ± SD (*n* = 4) of three independent experiments. MIC was expressed as the mean concentration between the well showing growth and that showing no growth. The MBC was expressed as the lowest concentration of the compounds showing no any bacterial growth after incubating for 20 h; * Significant difference (*p* < 0.01) compared the MIC values of compounds **1**–**4** with **5**–**9** (MIC > 16.0 mg·mL^−1^, data not shown), respectively.

## 3. Experimental Section 

### 3.1. General 

The NMR spectra were measured on Bruker AVANCE 400 MHz NMR instrument (Bruker SpectroSpin, Karlsruhe, Germany), and chemical shifts are given as δ (ppm) while the coupling constants are given in Hz. Xero Q Tof MS spectrometer (Waters, Milford, MA, USA) was used to measure and analysis the HRESIMS data. Volatile derivatives from compounds were analyzed on a GC-MS (Angilent, California, CA, USA) instrument. Waters 2535 instrument coupled with a Waters Sunfire prep C18 OBD (19 × 250 mm i.d.) column, a UV-2998 (Waters, MA, USA), and RI-2414 detector as a Preparative HPLC (Waters, MA, USA) was used to prepare compounds. FTIR-8400S (Shimadzu, Kyoto, Japan) was used to record the IR Spectra data; Column chromatographies including Macroporous resin (AB-8 Crosslinked Polystyrene, Nankai Chemical Plant, Tianjin, China), silica gel (200–300 mesh, Haiyang Chemical Group Co. Ltd., Qingdao, China), and ODS-A (120A, 50 mm; YMC, Kyoto, Japan) were also employed. A microplate reader (BMG FLUOStar OPTIMA, Ortenberg, Germany) was used to monitor the growth of the bacterial strains.

### 3.2. Bacterial Strains and the Preparation of Inoculums

The bacterial strains of *E. coli*(ATCC25922), *S. aureus* (ATCC25923), *S. flexneri* (ATCC12022), and *S. typhi* (ATCC14028) from American Type Culture Collection were provided by Department of Microbiology and Immunology, Heilongjiang university of Chinese medicine. Strains from refrigerated stock cultures were inoculated into common agar plate and incubated at 37 °C for 18 h. The bacteria were activated in nutrient broth and incubated at 37 °C for another 18 h. The concentrations of strains for antibacterial test *in vitro* were 5 × 10^5^ CFU·mL^−1^. 

### 3.3. Plant Materials 

We collected the aerial part of *P*. *oleracea* from the Dongfanghong Forestry Agency (Jixi, China) and identified by Lianjie Su of Heilongjiang University of Chinese Medicine. The voucher specimen (No. 20130814) is deposited at the Herbarium of Heilongjiang University of Chinese Medicine, China.

### 3.4. Extraction and Isolation 

The dried *P*. *oleracea* (15.0 kg) extracted with 70% EtOH under reflux (2 × 120 L) for 2 h. The 70% EtOH extract (1770 g) was suspended in H_2_O (20 L), and successively extracted with petroleum ether (60–90 °C), EtOAc and *n*-butanol, respectively. Solvent was removed to give petroleum ether extract (125.3 g), EtOAc extract (173.7 g), *n*-butanol extract (184.8 g), and remained water extract (1267.2 g). The EtOAc extract is the antibacterial effective fraction as previous study. The EtOAc fraction (150.0 g) was repeatedly column chromatographed on silica gel with a gradient of CH_2_Cl_2_/MeOH (40:1 to 0:1) as eluents to afford seven fractions: F_1_–F_7_. F_2_ (13.2 g) continued silica gel chromatography and eluted with petroleum ether/EtOAc (15:1 to 1:1) to afford sub-fractions A_1_–A_4_. Compounds **4** (53 mg) and **9** (37 mg) were obtained by silica gel chromatography of the sub-fraction A_2_ (3.5 g) elution with petroleum ether/EtOAc (8:1), F_3_ (25.7 g) was subjected to column chromatography on silica gel with CH_2_Cl_2_/MeOH (30:1 to 10:1) to afford sub-fractions B_1_–B_5_. B_3_ (6.5 g) was separated on silica gel chromatography and eluted with CH_2_Cl_2_/MeOH (40:1 to 15:1) and further purified by preparative HPLC on a Hypersil-ODS II column (10 μm, 20 × 300 mm, flow rate 8 mL/min) with MeOH/H_2_O (48% and 52%) to afford compound **5** (43 mg, *t*_R_ = 17 min), **7** (38 mg, *t*_R_ = 24 min), and **8** (33 mg, *t*_R_= 28 min). F_5_ (32.6 g) was subjected to column chromatography on silica gel with CH_2_Cl_2_/MeOH (30:1–8:1) to produce sub-fractions C_1_–C_5_. The C_3_ (4.0 g) was repeatedly column chromatographed on silica gel with CH_2_Cl_2_/MeOH (15:1 to 6:1) as eluents to afford **2** (40 mg) and **6** (21 mg). The C_4_ (5.9 g) was repeatedly column chromatographed on silica gel with CH_2_Cl_2_/MeOH (15:1 to 6:1) as eluents and followed separated on column chromatography Sephadex LH-20 with MeOH as eluent to afford **1** (32 mg) and **3** (37 mg).

*Portulacerebroside B* (**1**): white amorphous powder, ${\left[\alpha \right]}_{D}^{22}$ = +13.3° (*c* = 0.52, C_5_H_5_N); IR (KBr) ν_max_ 3420, 2933, 2837, 1645, 1542, 1471, 1280, 1158, 1082, 722 cm^−1^; ESIMS *m*/*z* 702 (100) [M + H]^+^; HRESIMS [M + H]^+^*m*/*z* 702.5516, calc. 702.5520 for C_39_H_75_NO_9_H; ^1^H- and ^13^C-NMR data, see Table 1 and Table 2.

*Portulacerebroside C* (**2**): white amorphous powder; ${\left[\alpha \right]}_{D}^{22}$ = +14.8° (*c* = 0.40, C_5_H_5_N); IR (KBr) ν_max_ 3405, 2927, 2832, 1634, 1536, 1468, 1155, 718 cm^−1^; ESIMS *m*/*z* 688 (100) [M + H]^+^; HRESIMS [M + H]^+^*m*/*z* 688.5358 calc. 688.5364 for C_38_H_73_NO_9_H; ^1^H- and ^13^C-NMR data, see Table 1 and Table 2.

*Portulacerebroside D* (**3**): white amorphous powder; ${\left[\alpha \right]}_{D}^{22}$ = +8.2° (*c* = 0.15, C_5_H_5_N); IR (KBr) ν_max_ 3412, 2941, 2838, 1638, 1530, 1455, 1162, 722 cm^−1^; ESIMS *m*/*z* 814 (100) [M + H]^+^; HRESIMS [M + H]^+^*m*/*z* 814.6765 calc. 814.6772 for C_57_H_91_NO_9_H; ^1^H- and ^13^C-NMR data, see Table 1 and Table 2.

*Portulaceramide A* (**4**): white amorphous powder; ${\left[\alpha \right]}_{D}^{22}$ = +9.5° (*c* = 0.30, C_5_H_5_N); IR (KBr) ν_max_ 3401, 3212, 2921, 1621, 1533, 1474, 723 cm^−1^; ESIMS *m*/*z* 472 (100) [M + H]^+^; HRESIMS [M + H]^+^*m*/*z* 472.3995 calc. 472.4002 for C_27_H_53_NO_5_H; ^1^H- and ^13^C-NMR data, see Table 1 and Table 2.

### 3.5. Methanolysis of **1**–**4**

Methanolysis experiment of **1** was conducted as previous studies [25,26]. Briefly, compound **1** (5.0 mg) was refluxed with 5% HCl in 82% aqueous MeOH (20 mL) for 18 h. After that the reaction mixture was extracted with *n*-hexane and the fatty acid methyl ester (FAME) was obtained as a white amorphous powder, ${\left[\alpha \right]}_{D}^{22}$ = −5.8° (*c* 0.02, CHCl_3_). The FAME was analyzed by GC-MS and the characteristic fragment ions (*m*/*z* 286 [M]^+^, 228 [M − COOMe]^+^) were obtained. Therefore, the FAME of **1** was determined as 2*R*-hydroxypentadecanoic acid methyl ester. The monosaccharide of **1** was determined as d-glucose (*t*_R_ = 7.26 min) by analyzing the remained solution on a GC-MS. The remained solution was evaporated MeOH followed by adjusting pH 9 with aqueous ammonia. The solution was extracted with Et_2_O and the Et_2_O layer was dried to obtain the long-chain base (LCB) of **1**. The LCB was analyzed by ESIMS to obtain the fragment ions of *m*/*z* 300 [M + H]^+^ and 282 [M − H_2_O + H]^+^. Thus, the LCB of **1** was determined as 2-aminooctadec-8-ene-1,3-diol (Figure 1). 

Methanolysis of **2** was performed by the same method for **1** and an FAME and an LCB was obtained respectively. The FAME was a white amorphous powder, ${\left[\alpha \right]}_{D}^{22}$ =−6.8° (*c* 0.03, CHCl_3_), and the characteristic fragment ions (*m*/*z* 286 [M]^+^, 228 [M − COOMe]^+^) were obtained by GC-MS analysis. Therefore, the FAME of **2** was determined as 2*R*-hydroxypentadecanoic acid methyl ester. The monosaccharide of **2** was also determined as d-glucose. The LCB of **2** was analyzed by ESIMS to obtain the fragment ions of *m*/*z* 286 [M + H]^+^ and 268 [M − H_2_O + H]^+^. Thus, the LCB of **2** was determined as 2-aminoheptadecenoic-4-ene-1,3-diol (Figure 1).

Methanolysis of **3** was performed by the same method for **1** and an FAME and an LCB was also obtained respectively. The FAME was a white amorphous powder, ${\left[\alpha \right]}_{D}^{22}$ = −3.8° (*c* 0.02, CHCl_3_), and the characteristic fragment ions (*m*/*z* 384 [M]^+^, 326 [M − COOMe]^+^) were obtained by GC-MS analysis. Therefore, the FAME of **3** was determined as 2*R*-hydroxydocosanoic acid methyl ester. The monosaccharide of **3** was also determined as d-glucose. The LCB of **3** was analyzed by ESIMS to obtain the fragment ions of *m*/*z* 314 [M + H]^+^ and 296 [M − H_2_O + H]^+^. Thus, the LCB of **3** was determined as 2-aminononadecane-4-ene-1,3-diol (Figure 1).

Methanolysis of **4** was also performed to obtain the FAME and LCB. The FAME was a white amorphous powder, ${\left[\alpha \right]}_{D}^{22}$ = −4.7° (*c* 0.02, CHCl_3_), and the characteristic fragment ions (*m*/*z* 286 [M]^+^, 228 [M − COOMe]^+^) were obtained by GC-MS analysis. Therefore, the FAME of **4** was determined as 2*R*-hydroxypentadecanoic acid methyl ester. The LCB of **4** was analyzed by ESIMS to obtain the fragment ions of *m*/*z* 232 [M + H]^+^, 214 [M − H_2_O + H]^+^, 196 [M − 2H_2_O + H]^+^ and 178 [M − 3H_2_O + H]^+^. Thus, the LCB of **4** was determined as 2-aminododecane-8-ene-1,3,4-triol (Figure 1).

### 3.6. Dimethyl Disulfide Derivative of LCBs from ***1*** and ***4***

According to the reference [18], LCBs from **1** and **4** (0.5 mg) were dissolved in dimethyl disulfide (DMDS, 0.2 mL), respectively, and then iodine (1 mg) was added into the solutions. The mixtures were stored in a small-volume sealed vial at 60 °C for 40 h. The reaction was ended with aqueous Na_2_S_2_O_3_ (5%), and then we extracted the mixtures with *n*-hexane (0.3 mL). The extracts were concentrated respectively to give the DMDS derivatives of LCBs from **1** and **4**. The derivatives were analyzed by ESIMS. As a result, the characteristic fragment ions of *m/z* 187 [M + H]^+^ for **1** and 117 [M + H]^+^ for **4** were observed respectively.

### 3.7. Antibacterial Test in Vitro

#### 3.7.1. Compounds **1**–**9** Serial Dilution

A microdilution method was used to determine the MICs of the compounds on 96-well cultivated plates according to the previous report [27]. The compounds **1**–**9** were dissolved in nutrient broth with 10% DMSO and 32.0 mg·mL^−1^ solutions (pH 7.2) were obtained, respectively. There were 12 wells in each row of a microplate, to each of the first ones we added 100 μL compound solution, and to the remaining 11 wells we added 100 μL broth culture. For serial dilution, 100 μL each compound solution was added into the second well and then 100 μL was sequentially transferred to the following wells until the 10th well. The last two wells served as growth control and sterility check. After that, 100 μL of inoculum was added into each well except the last well in which 100 μL broth was added instead. Amoxicillin was used as a positive drug.

#### 3.7.2. Determination of MICs and MBCs

The growth of the bacterial strains in the microplates was monitored at 37 °C for 20 h using a microplate reader. Standard antibacterial agent amoxicillin was also screened under identical conditions for comparison. Considering the role of DMSO, the same experiment was carried out with 10% DMSO and showed no activity against any bacterial strains. MIC was expressed as the mean concentration between the well showing growth and that showing no growth. 

After MIC testing, the microplates set up for the MICs determination were used to determine the MBC as described previously [28]. For each well showing no bacterial growth, the entire volume was spread onto nutrient agar plates and subcultured. The MBC was defined as the lowest concentration of the compounds showing no bacterial growth after incubating for 20 h.

## 4. Conclusions 

We investigated the chemical constituents of *P*. *oleracea* based on its antibacterial activity and nine compounds were obtained, including three new cerebrosides and a new ceramide. The structures of new compounds were identified as 1-*O*-β-d-glucopyranosyl-(2*S*,3*R*,8*E*)-2-[(2′*R*)-2-hydroxylpentadecanoylamino]-8-octadecene-1,3-diol (**1**), 1-*O*-β-d-glucopyranosyl-(2*S*,3*R*,4*E*)-2-[(2′*R*)-2-hydroxylpentadecanoyl-amino]-4-heptadecene-1,3-diol (**2**), 1-*O*-β-d-glucopyranosyl-(2*S*,3*R*,4*E*)-2-[(2′*R*)-2-hydroxyl-docosanamideamino]-16-methyl-4-nonadecene-1,3-diol (**3**), and (2*S*,3*S*,4*R*,8*E*)-2-[(2′*R*)-2-hydroxyl-pentadecanoylamino]-8-dodecene-1,3,4-triol (**4**), respectively. Antibacterial tests *in vitro* showed that new compounds could significantly inhibit or kill the common enteropathogenic bacteria, which might contribute to revealing the usefullness of *P*. *oleracea* as a treatment for bacillary dysentery.
